# Cascaded domain multiphoton spatial frequency modulation imaging

**DOI:** 10.1117/1.JBO.28.10.106502

**Published:** 2023-10-04

**Authors:** Daniel Scarbrough, Anna Thomas, Jeff Field, Randy Bartels, Jeff Squier

**Affiliations:** a Colorado School of Mines, Department of Physics, Golden, Colorado, United States; b Colorado State University, Department of Electrical and Computer Engineering, Fort Collins, Colorado, United States; c Colorado State University, Center for Imaging and Surface Science, Fort Collins, Colorado, United States; d Colorado State University, School of Biomedical Engineering, Fort Collins, Colorado, United States

**Keywords:** microscopy, structured illumination, multiphoton, enhanced resolution, spatial frequency modulation imaging, dispersion compensation, single pixel detection

## Abstract

**Significance:**

Multiphoton microscopy is a powerful imaging tool for biomedical applications. A variety of techniques and respective benefits exist for multiphoton microscopy, but an enhanced resolution is especially desired. Additionally multiphoton microscopy requires ultrafast pulses for excitation, so optimization of the pulse duration at the sample is critical for strong signals.

**Aim:**

We aim to perform enhanced resolution imaging that is robust to scattering using a structured illumination technique while also providing a rapid and easily repeatable means to optimize group delay dispersion (GDD) compensation through to the sample.

**Approach:**

Spatial frequency modulation imaging (SPIFI) is used in two domains: the spatial domain (SD) and the wavelength domain (WD). The WD-SPIFI system is an in-line tool enabling GDD optimization that considers all material through to the sample. The SD-SPIFI system follows and enables enhanced resolution imaging.

**Results:**

The WD-SPIFI dispersion optimization performance is confirmed with independent pulse characterization, enabling rapid optimization of pulses for imaging with the SD-SPIFI system. The SD-SPIFI system demonstrates enhanced resolution imaging without the use of photon counting enabled by signal to noise improvements due to the WD-SPIFI system.

**Conclusions:**

Implementing SPIFI in-line in two domains enables full-path dispersion compensation optimization through to the sample for enhanced resolution multiphoton microscopy.

## 1 Introduction

Since the introduction of confocal microscopy in 1961,^1^ it has become a mainstay in biological studies and continues to evolve today with numerous advancements. One such recent advancement by Abouakil et al. implements a multi-step scanning process with an algorithm to identify structures of focus for finer scanning,^2^ enabling faster confocal imaging of specific structures of interest. Another development by Berlage et. al. makes use of three-photon excitation to penetrate deeper into biological tissue and uses adaptive point spread function (PSF) engineering to reduce scattering effects.^3^ Of particular interest, however, is studying features beyond the standard resolution limit to further our understanding of cell dynamics and related biological processes. For example, Wu et. al. presented a technique using a multi-view approach^4^ to break this limit. Their technique uses multiple line cursors through three objectives and deep learning to achieve enhanced resolution.

As enhanced resolution enables the study of ever smaller structures, the field of structured illumination microscopy (SIM) is developing with a multitude of imaging techniques. Since the early conception of SIM by Köhler,^5^ SIM has taken many forms that enable enhanced resolution. The HiLo technique,^6^^,^^7^ for example, has been extended to light sheet fluorescence microscopy,^8^ multiphoton temporal focusing,^9^ and line scanning.^10^ Other forms of SIM have been developed to achieve enhanced resolution using techniques such as interferometry,^11^ intensity modulation,^12^ fiber arrays,^13^ and temporal encoding.^14^ Recent computational advances have enabled deep learning to be applied to standard commercial SIM instruments to improve resolution.^15^ Common among these techniques is the use of 2D array detectors [charge-coupled device (CCD) and complementary metal oxide semiconductor (CMOS)] or modulation devices, such as spatial light modulators or digital micromirror devices (DMDs). Within SIM, there is also significant effort being put forth to make use of simpler and more cost-effective modulation schemes and detectors while still achieving enhanced resolution. As part of this, using single-pixel detectors is of interest, especially for low-light applications such as multiphoton microscopy.

Single pixel detection pairs well with SIM as the structure enables multidimensional image reconstruction despite the zero-dimensional detector. Compressive sensing with an L1 norm minimization has achieved 100 frames per second (fps),^16^ and others have gone to kHz rates using acousto optic modulators.^17^ Multiphoton compatible techniques have also been demonstrated using temporal focusing from a DMD,^18^ and another technique uses a more cost effective spinning mask modulation for Hadamard reconstruction.^19^

### 1.1 Spatial Frequency Modulation Imaging

One structured illumination single pixel detection technique that is also capable of enhanced resolution is spatIal frequency modulation imaging (SPIFI). SPIFI encodes temporal frequency information onto the spatial domain (SD) of a line cursor. Commonly this encoding is imparted via a rotating reticle on which a pattern based on the Lovell electro-optical position indicator^20^ is printed. This pattern has a frequency modulation that changes linearly with the radius^21^ in a spinning disk modulation scheme. As this modulation mask rotates, each pixel along the line cursor is modulated at a unique temporal frequency. This mapping from the SD to a temporal frequency domain enables single element detection by measuring the collected signal in time and reconstructing the image data with a fast Fourier transform (FFT). This structured, single-pixel detection approach mitigates the effects of scattering through a diffusive sample that typically blurs an image captured by a 2D detector array. Because SPIFI uses an FFT reconstruction, it maintains a low reconstruction complexity of O(MN log N), where M is the number of line images and N is the number of points transformed by the FFT (one line exposure). Additionally, with the line scanning inherent to SPIFI, pixel dwell time is improved over point rasterization techniques. For example, a 256×256  pixel image taken at 30 fps with a point scanning technique has only 510 ns of exposure per pixel. For multiphoton imaging with a pulsed laser at a repetition rate of 100 MHz, this would result in 50 pulses being incident on each pixel. However, for a line scanning architecture in which the line is scanned across 256 steps, a 30 fps image can be taken with a pixel dwell time of 65  μs and 6510 pulses per pixel on a mask with a 50% duty cycle.

Since SPIFI was first introduced as an imaging technique,^22^ it has been demonstrated and extended to multiple imaging modalities including fluorescent imaging,^22^^,^^23^ multiphoton excitation fluorescence imaging,^24^^–^^26^ second harmonic generation imaging,^26^^,^^27^ phase imaging,^28^^,^^29^ and localization microscopy.^30^ Extending SPIFI to provide simultaneous SIM benefits in two dimensions has also been explored using 2D modulation schemes,^31^^,^^32^ random-access imaging,^33^ parallel line image acquisition,^34^ single pixel tomographic imaging,^35^^–^^38^ multi-cursor 2D imaging,^32^ random access multiphoton imaging,^33^ and coherent anti-Stokes Raman spectroscopy.^27^

Here we introduce a system that employs SPIFI in two domains to (1) achieve enhanced resolution multiphoton microscopy and (2) compensate for second order dispersion with a rapid, facile, and easily repeatable method. Significantly, the presented dispersion compensation method accounts for second order dispersion all the way through to the sample plane, providing an in-line technique that considers the full path of the microscope and requires no diversion of the beam. The heart of the SPIFI system in either the wavelength domain (WD) or the SD is the SPIFI modulation mask, which is modeled in polar coordinates for a disk reticle as

$$M[r,\theta (t)]=\frac{1}{2}\{1+\cos[k(r_{0}+r)\theta (t)]\},$$ (1)

where r is the radius on the mask and θ(t) is the rotation of the mask through time. The spatial frequency of the mask pattern at each angle is determined by the chirp rate k and an offset $r_{0}$. At each position of r, a unique temporal frequency is encoded throughout the mask rotation determined by $k(r_{0}+r)$. This encoding enables image reconstruction via an FFT of the time signal. Here we summarize the analysis of the SPIFI signals presented in Refs. 26 and 38 and account for second order multiphoton imaging as in two-photon excitation fluorescence (2PEF) or second harmonic generation (SHG). For simplicity, the mask equation is written with a time dependent spatial frequency $f_{r}(t)$ as

$$M(r,t)=\frac{1}{2}\{1+\cos[2\pi f_{r}(t)r]\}.$$ (2)

Focusing the field along one mask radius E(r) ensures that each “pixel” along the line cursor is modulated at one frequency. The modulated field is then imaged to an object c(r), resulting in E(r)M(r,t)c(r). A detector measures the intensity of this field, so it is squared: E(r) becomes I(r), M(r,t) is simplified with a trigonometric reduction using the identity $\cos^{2}(\theta )=1/2[1+\cos(2\theta )]$, and $c^{2}(r)$ is written as C(r) for simplicity. For a process such as 2PEF or SHG, the signal light of interest is dependent on the intensity squared. As an example, for an object imaged with 2PEF, the object being imaged is written as $C_{2P}(r)$, representing its two-photon spatial response. As such, the measured intensity ($I_{m}$) for linear imaging modalities and second order multiphoton modalities are respectively written as

$$I_{m}(r,t)=I(r)\frac{1}{8}\{3+4\;\cos[2\pi f_{r}(t)r]+\cos[2\pi 2f_{r}(t)r]\}C(r),$$ (3)

$$I_{m}^{2}(r,t)=I^{2}(r)\frac{1}{128}\{35+56\;\cos[2\pi f_{r}(t)r]+28\;\cos[2\pi 2f_{r}(t)r]\;+8\;\cos[2\pi 3f_{r}(t)r]+\cos[2\pi 4f_{r}(t)r]\}C_{2P}(r).$$ (4)

Each of these terms in the intensity can be examined individually to analyze the detected signal. As the single element detector spatially integrates the full intensity signal, we can look at the integral of each term independently. For convenience, the integral is examined separately for each of the non-constant terms in $I_{m}^{2}$ from Eq. (4). The leading constants and time dependent amplitude (due to modulation and vignetting) are wrapped into $H_{q}(t)$, where q represents the “order” of the term.^26^ Expanding these terms and simplifying with Euler’s formula result in a Fourier transform. The factors of 1, 2, 3, and 4 on $f_{r}(t)$ are carried through into the Fourier transform of the object’s 2P response, yielding four equations with increasing spatial frequency support:

$$S_{\pm 1}(t)=H_{1}(t)\int_{-\infty }^{\infty }dr\;\exp[\pm i2\pi f_{r}(t)r]C_{2P}(r)=H_{1}(t){\overset{˜}{C}}_{2P}[\pm f_{r}(t)],$$ (5)

$$S_{\pm 2}(t)=H_{2}(t)\int_{-\infty }^{\infty }dr\;\exp[\pm i2\pi 2f_{r}(t)r]C_{2P}(r)=H_{2}(t){\overset{˜}{C}}_{2P}[\pm 2f_{r}(t)],$$ (6)

$$S_{\pm 2}(t)=H_{3}(t)\int_{-\infty }^{\infty }dr\;\exp[\pm i2\pi 3f_{r}(t)r]C_{2P}(r)=H_{3}(t){\overset{˜}{C}}_{2P}[\pm 3f_{r}(t)],$$ (7)

$$S_{\pm 4}(t)=H_{4}(t)\int_{-\infty }^{\infty }dr\;\exp[\pm i2\pi 4f_{r}(t)r]C_{2P}(r)=H_{4}(t){\overset{˜}{C}}_{2P}[\pm 4f_{r}(t)].$$ (8)

**Fig. 1:**
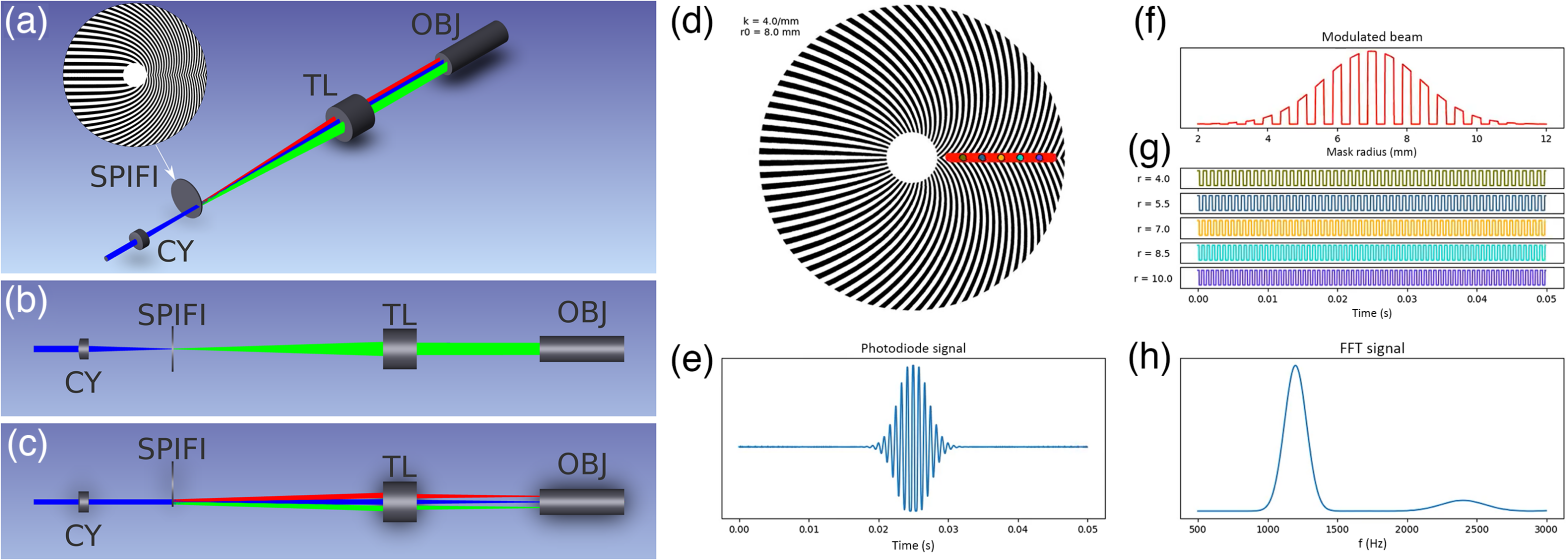
(a) Isometric view of a SPIFI microscope. CY, cylindrical lens; SPIFI, SPIFI mask; TL, tube lens; and OBJ, objective. (b) X-Z view of SPIFI microscope and (c) Y-Z view of SPIFI microscope. This view makes clear the line focus on the SPIFI mask, the diffracted orders from the SPIFI mask, and the spread of the diffracted orders on the objective pupil. (d) Simulated SPIFI mask with parameters $k=4.0\;{\mathrm{mm}}^{-1}$ and $r_{0}=8.0$. The red line represents the focused line beam, and the colored dots along it correspond to single pixels on the line with modulations that are shown in panel (g). (e) Simulated time signal from a photodiode. This is the spatial integral along the line cursor at each time step, corresponding to one rotation step. No object is present in this simulation. (f) Intensity profile of the line cursor at one modulation time step. (g) Normalized modulation of each colored pixel highlighted in panel (d). Each pixel has a unique temporal frequency, which enables its extraction into a line image via FFT. (h) FFT of the SPIFI trace in panel (e), which shows the first and second order images. Note the second order is centered at twice the frequency of the first order, the amplitude is lower, and the width of the second order is twice that of the first. Panels (d)–(h) of this figure are featured from a SPIFI simulation with a video supplement available online (Video 1, MP4, 9.1 MB [URL: https://doi.org/10.1117/1.JBO.28.10.106502.s1]).

The increasing spatial frequency terms resulting from the Fourier transform is indicative of an increase in resolution. The signal equations can then be simply written for any value q, and only the positive portion of the Fourier Transform is needed. A diagram of a SPIFI system is shown in Figs. 1(a)–1(c). Figures 1(d)–1(h) show an example SPIFI mask, a modulated beam at one time step, the variations in temporal frequency modulation as a function of radial position, an example signal, and the resulting FFT reconstruction of the illumination beam. These portions of the figure are from a video to aid in understanding SPIFI and are included as a supplement to this article available online (Video 1).

**Fig. 2:**
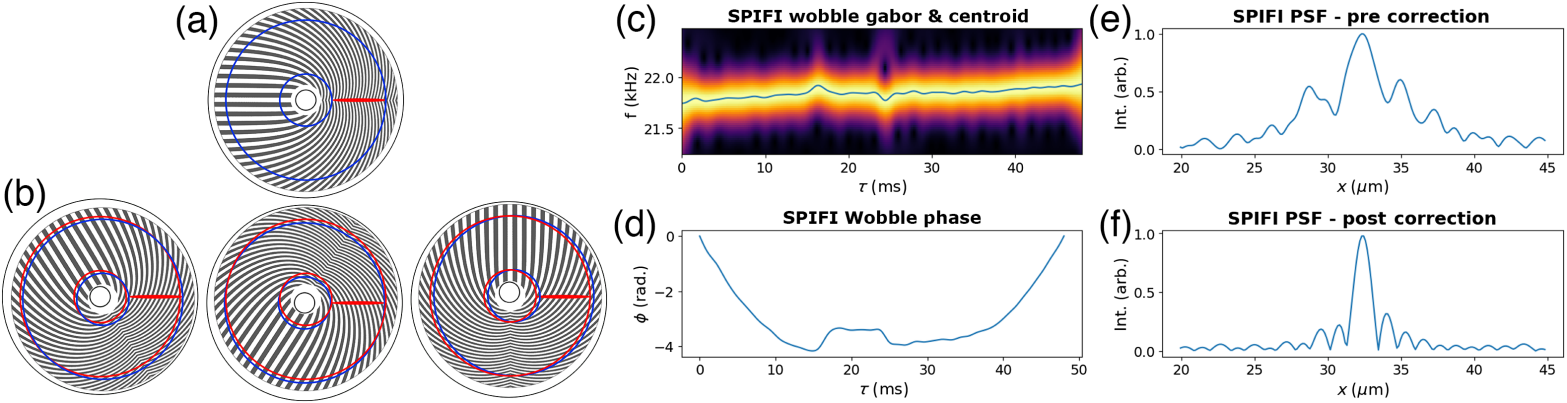
(a) A perfectly centered mounted SPIFI mask corresponding to a wobble phase of ϕ(t)=0. The blue lines show that the leftmost and rightmost pixels along the line cursor maintain a constant radius from the center of the mask. (b) An off-center mask at three rotation angles. The red circles show the varying radii from the true center of the mask, yielding a wobble phase. (c) A Gabor transform of first order SPIFI data showing the central modulation frequency shifting through time. The blue line is the centroid. (d) The wobble phase obtained via a cumulative integral of the centroid in panel (c). (e) The PSF prior to wobble correction. (f) The PSF following wobble correction.

An additional factor to consider is that the SPIFI mask rotates at a temporal frequency $\nu_{c}$, and this effect needs to be included in the time signal. Another consideration is that, if the mask is not perfectly mounted on the center of the rotation axis, the modulation frequencies will “wobble” throughout rotation. An example of this is shown in Fig. 2. This wobble is represented by a phase term with time dependence ϕ(t). This phase term can be extracted by analyzing the SPIFI carrier frequency $\nu_{c}$ throughout rotation with a stepped windowed Fourier transform known as the Gabor transform. Including both the wobble and carrier frequency in the analysis starting again from Eq. (2) yields a new form of the signal equation $S_{q}(t)$ that depends on these factors for each order; it is written as

Sq(t)=Hq(t)Re{exp[i2πqνct]exp[iqϕ(t)]C˜2P[qfr(t)]}. (9)

The result is a signal with multiple orders separated by the carrier frequency $q\nu_{c}$ and wobbled by qϕ(t), with increasing spatial frequency support $qf_{r}(t)$. The tradeoff of the higher orders in SPIFI is the reduced signal amplitudes in $H_{q}(t)$. To maintain fidelity in the higher order images, SNR has to be maximized to account for these lower amplitudes. Signal averaging over longer exposure times can improve the SNR, but in multiphoton implementations, averaging the raw PMT signals is not viable. However, with SPIFI, FFT images can be generated from raw PMT signals, and those FFT images can be averaged. All results presented in this paper are processed in this way; however, further SNR improvements may be made using photon counting to build up the SPIFI signal over time with discrete time bins counting the detected events corresponding to the excitation laser pulse arrivals.

#### 1.1.1 SPIFI wobble correction

Spinning disk structured illumination techniques are sensitive to variations due to the modulation center being offset from the rotational center due to mounting onto the motor axle being used. Some techniques, such as the spinning Hadamard technique,^19^ have adjusted for this by scanning the illumination source synchronously with the disk to eliminate this effect. Because each radial position in SPIFI is encoded with a temporal frequency, the mask wobble manifests as a frequency phase that shifts throughout the rotation,^26^ enabling measurement and correction without scanning of the illumination source.

The wobble of a SPIFI system can be measured once for a system and applied as a correction for all subsequent measurements. To do so, the frequency shift throughout rotation is measured by imaging a single small object, such as a pinhole or fluorescent particle smaller than the PSF. With only one “pixel” on the line cursor yielding the collected signal light, the frequency encoding of SPIFI would ideally result in a single temporal frequency captured by the detector. With wobble present, the shifting frequencies throughout rotation effectively blur the PSF and in turn blur the reconstructed images.

To characterize wobble, the first order SPIFI signal (of size N) is isolated in the FFT by selecting its region in frequency space and setting all other values to zero (size N). Taking the IFFT of this results in a temporal signal (of size N) containing only the signal information corresponding to the first order frequency modulation [Eq. (9) with q=1]. In a perfect system, this would be a sinusoid for the single modulation frequency of one pixel on the SPIFI cursor. With wobble, the signal shifts through multiple frequencies throughout the rotation. This shift can be measured with a Gabor transform—a sliding window Fourier transform across the isolated first order time signal. Using a Gaussian shape as the sliding window results in the transform

$$G_{x}(\tau ,f)=\int_{-\infty }^{\infty }\exp[-4\;\ln\;2{\left(\frac{t-\tau }{w_{g}}\right)}^{2}]x(t)dt,$$ (10)

where τ is the time grid through which the window slides and is determined by choosing the number of steps taken for a discrete Gabor transform, t is the original signal time grid, $w_{g}$ is the width of the Gaussian window in time, and x(t) is the signal to transform. The width of the Gaussian is typically set to ∼8−16x, the time step of the carrier frequency; this is a general rule of thumb, but not a hard rule, that often produces an effective wobble measurement. Larger window sizes increase the time domain resolution but reduce the resolution in the frequency domain, and vice-versa for smaller window sizes. The factor of 4 ln 2 adjusts the Gaussian window such that the full width at half max (FWHM) is near the desired $w_{g}$. An additional variable to consider adjusting is the number of steps in τ, which can go up to the number of data points in t (without extra interpolation). Doing so increases the time domain resolution and the computation time. An example spectrogram with its centroid and the resulting phase is shown in Fig. 2. These data were taken with 2PEF contrast from a sub-diffractive nano-diamond, which is effectively a measure of the system’s PSF.

In the spectrogram, looking at the frequency region for first order SPIFI shows the frequency shifting throughout the mask rotation. Calculating the centroid of the spectrogram and then taking a cumulative integral relative to the mean of the centroid and multiplying by 2π yields a phase shift with time dependence ϕ(τ). This phase can be interpolated onto the time grid of any subsequent measurement for correction as ϕ(t). Correction is applied by again isolating the order of interest signal in the FFT (set all values outside the region of interest to zero), taking the IFFT, multiplying the result by exp[−iqϕ(t)], and then taking the FFT again to get the corrected image. This correction effectively narrows the PSF for each SPIFI order.^39^ The phase, uncorrected image, and corrected image of a fluorescent nanodiamond are shown along with the spectrogram in Fig. 2. For orders beyond the first, the same wobble phase is applied with a factor for the order q as in Eq. (9). As an example, to correct the second order SPIFI image, the second order would be isolated, the IFFT taken, the correction exp[−i2ϕ(t)] applied, and the FFT taken again for the corrected image.

### 1.2 Wavelength Domain and Spatial Domain SPIFI Microscopy

**Fig. 3:**
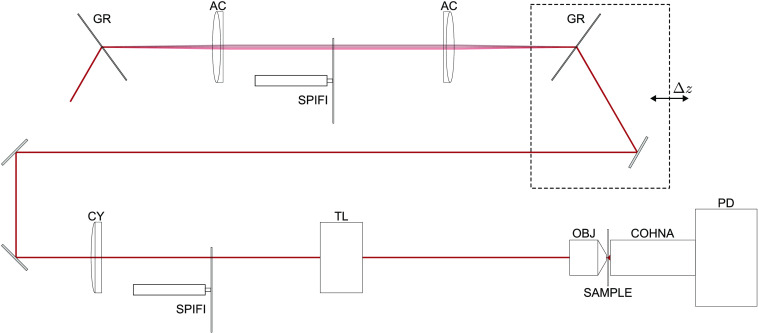
Diagram of the cascaded WD-SD-SPIFI system. The WD-SPIFI subsystem includes the first grating through the mirror following the second grating. The SD-SPIFI subsystem is as shown in Fig. 1, starting from the cylindrical lens. The collection optics and detector are also shown in this diagram. GR, grating; AC, achromat; SPIFI, SPIFI mask; Δz, grating offset distance from lens f; CY, cylindrical lens; TL, tube lens; OBJ, objective lens; COHNA, collection optic with high numerical aperture;^40^ and PD, photodetector (photodiode or photomultiplier tube).

In this paper, we present an advancement in SPIFI multiphoton microscopy by cascading SPIFI modulation in two domains. First is a WD SPIFI (WD-SPIFI) subsystem for dispersion compensation optimization, followed by a standard SD SPIFI (SD-SPIFI) subsystem for imaging. The full cascaded system is shown in Fig. 3. The WD-SPIFI system is a modification from previous work in which a scanning slit is used in a Martinez pulse compressor to characterize the laser pulse.^41^ By switching the scanning slit to a rotating SPIFI mask, diffracted orders from the SPIFI mask are generated, yielding copies of the pulse with a relative sweeping time delay. The relative delay is varied as the mask rotates due to the diffraction from the SPIFI reticle, which changes as a function of the mask angle. This effect is utilized using a specimen that responds nonlinearly to the structured excitation pulses, e.g., either 2PEF or SHG. Moving the output grating of the WD-SPIFI system adds a negative group delay dispersion (GDD), which can be used to compensate for the positive GDD imposed by the downstream glass in the SD-SPIFI microscope.

**Fig. 4:**
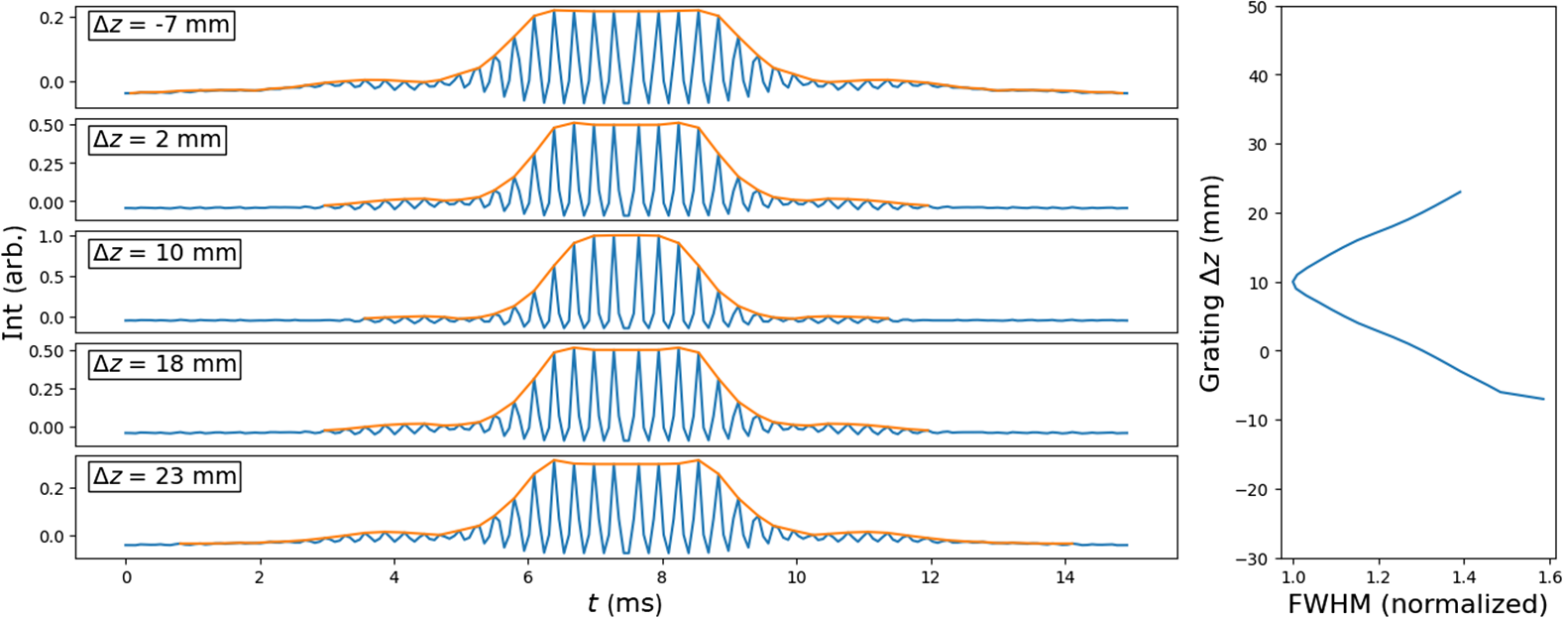
Left: simulated second order multiphoton signals of the WD-SD-SPIFI dispersion optimization system at varying output grating offsets. The shown envelope profiles are determined using a peak-detection algorithm on the signals. Right: FWHM of the signal envelopes for each grating position. The signal with the highest relative intensity also yields the shortest relative pulse width (Δz=10  mm). The plot range is extended along Δz to be consistent with following results that use WD-SPIFI with dispersion scan.

The WD-SPIFI signals for the dispersion optimization system were simulated in Zemax at varying output grating offsets. The results are shown in Fig. 4. This simulation produced nonlinear intensity signals throughout a SPIFI mask rotation through a range of grating positions. For each grating position, an envelope can be drawn from the peaks of the signal. The FWHM of each signal can be measured from these envelopes, and it is found that the shortest FWHM corresponds to the grating position that best compensates for the simulated downstream dispersion. As shown by the simulation, the WD-SPIFI system enables a straightforward method for optimizing GDD compensation by simply observing the resulting signals’ FWHMs to determine the optimal grating position.

## 2 Optical System

**Fig. 5:**
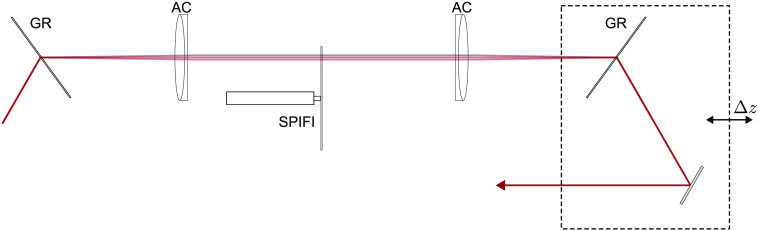
The WD-SPIFI system is a Martinez pulse compressor with a SPIFI mask at focus between the lenses. Adding an offset to the output grating past focus adds negative GDD. Note that the output grating is coupled with a mirror that reflects the beam parallel to the beam through the lenses to maintain alignment downstream through grating motion. GR, grating; AC, achromat; SPIFI, SPIFI mask on motor; and Δz, grating offset distance from lens f.

A Thorlabs FSL1030X1 source ($\lambda_{0}=1035\;\mathrm{nm}$, Δt≤220  fs, and f=10  MHz) was used to test the cascaded WD-SD-SPIFI system. The WD-SPIFI subsystem was built into a Martinez dispersion compensation system with two gratings (Lightsmyth T-1000-1040-31.8 × 24.8-94) at Littrow configuration and two lenses (Thorlabs AC508-100-B-ML) with all components initially in the 4f configuration. The WD-SPIFI system was built on a rail with carriers (Newport PRL-24 and PRC-3) for easy adjustment of the spacing of the second grating from the second lens. A SPIFI mask machined onto a glass disk using an in-house laser machining system^42^ (minimum line feature width: 180  μm) was mounted to a stepper motor (Trinamic QSH2818) and placed at f between both lenses. Coupling the output grating translation with a mirror that reflects the output parallel to the translation direction is critical for maintaining downstream alignment through translation. A schematic of the WD-SPIFI system is shown in Fig. 5, and experimental results for finding the optimal grating position for dispersion compensation are shown in Fig. 3.

The SD-SPIFI subsystem follows the WD-SPIFI subsystem and focuses the beam into a line cursor with a cylindrical lens (Thorlabs LJ1567L1-B) onto a SPIFI mask. The SPIFI mask was manufactured by InLight Gobo with a pattern line width of ∼30  μm and was mounted onto a motor (Faulhaber Minimotor SA). The exposure time for a single line image without averaging was ∼15  ms. An inconsistent motor speed is apparent; this modulates the carrier frequency $\nu_{c}$, blurring images from trace to trace. This is mitigated by monitoring a separate laser focused on the SPIFI mask. The signal from this timing laser enables consistent oscilloscope triggering and measuring of the duration between timing pulses on the SPIFI mask, which enables signal interpolation onto a common time grid (and thus common $\nu_{c}$). It is important that this laser is selected at a wavelength that is not near any of the fluorescent wavelengths, so it is filtered from the detector.

**Fig. 6:**
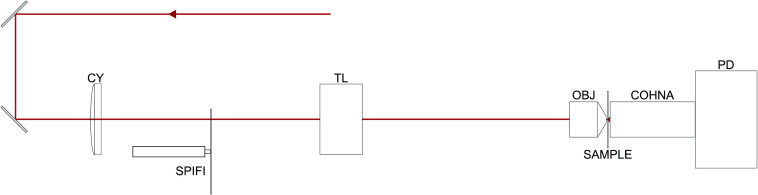
Diagram of the SD-SPIFI system. The reflected light from the mirror coupled with the translating WD-SPIFI output grating is sent through a spinning disk SPIFI microscope. CY, cylindrical lens; TL, tube lens; OBJ, objective lens; COHNA, collection optic with high numerical aperture;^40^ and PD, photodetector (photodiode or photomultiplier tube).

The SPIFI modulation plane was imaged to the sample plane through a tube lens (Thorlabs TTL200MP) and microscope objective (Olympus UPLFLN 40x). A collection optic with a high numerical aperture (COHNA^40^) with built-in space for an interchangeable filter was placed after the sample to collect multiphoton signal light onto a photomultipler tube (PMT - Hammamatsu H7422P-40). The output signal was transmitted via double shielded SMA cable (Pasternack PE3M0034) to a transimpedance amplifier (Thorlabs TIA60) and measured with an oscilloscope terminated on 50 Ohms (Digilent Analog Discovery Pro ADP3450) using a custom Python script. This script handles the acquisition as well as the scan control, communicating serially with a motor controller and stepper motors (Newport ESP301 and Newport LTA-HS) (Fig. 6).

## 3 System Performance

### 3.1 WD-SPIFI Performance

**Fig. 7:**
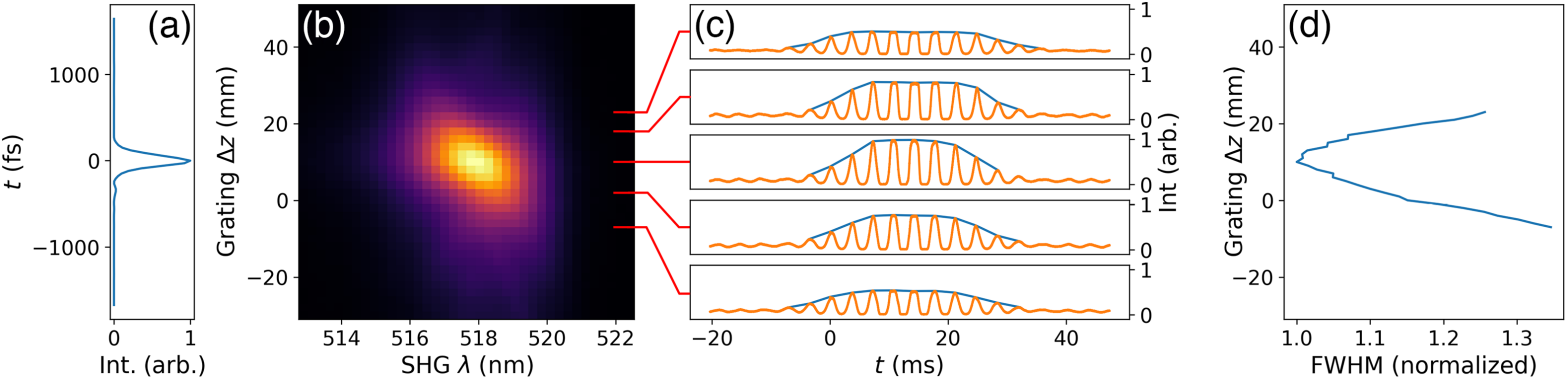
(a) Temporal pulse profile retrieved from dispersion scan. (b) Dispersion scan measurement showing spectral intensity at varying grating positions. (c) WD-SPIFI traces with envelopes at grating positions corresponding to those in dispersion scan. (d) FWHM of envelopes in WD-SPIFI as a function of the grating position.

The WD-SPIFI subsystem was used to optimize the dispersion compensation of the Martinez system by setting the position of the output grating according to the FWHM of the nonlinear signal generated by a 2PEF dye at the focus of the microscope objective. Initially, this was done with another microscope objective (Zeiss A-Plan 40x). The output grating was scanned in steps of 1 mm starting from a distance of −7  mm from the zero dispersion position to +23  mm past the zero dispersion point. At each grating position, a time trace over the full rotation of the WD-SPIFI mask was taken with 30 sample averages. The peaks of the traces were used to draw an envelope, and the FWHM of each was found. The results are shown in Fig. 7, which shows an optimal point at the lowest FWHM of the WD-SPIFI trace at a grating offset of +10  mm. This point indicates the optimal dispersion compensation as it corresponds to the shortest pulses at focus. This result was compared against the full pulse characterization technique, dispersion scan.^43^^–^^45^ The dispersion scan was taken over the same grating positions and shows a peak intensity at the same grating position as found by the WD-SPIFI optimization. Running the dispersion scan through its iterative phase retrieval algorithm showed a temporal pulse width of 209 fs and confirms the optimal location of the grating found in WD-SPIFI.

The WD-SPIFI optimization system allows for flexibility in the optical system without time-intensive re-characterization. For the following SD-SPIFI resolution results, the objective was changed back to the Olympus UPlan FLN 40x objective, the cylindrical lens was changed to a 50 mm achromatic cylindrical lens (ThorLabs ACY254-050-B), a beam expander prior to the SD-SPIFI system was removed, and the internal dispersion compensation of the ThorLabs FSL laser was reduced to 0. With a quick GDD compensation recalibration using the WD-SPIFI system, the offset of the grating was increased to +16 mm by again solely observing the signals displayed on an oscilloscope.

### 3.2 SD-SPIFI System Performance

In the SD-SPIFI subsystem, we show that the resolution characterization is possible both by measuring the PSF via imaging a sub-diffractive object and by measuring the modulation transfer function (MTF) directly using the spatial frequencies of the mask imaged through the system.

**Fig. 8:**
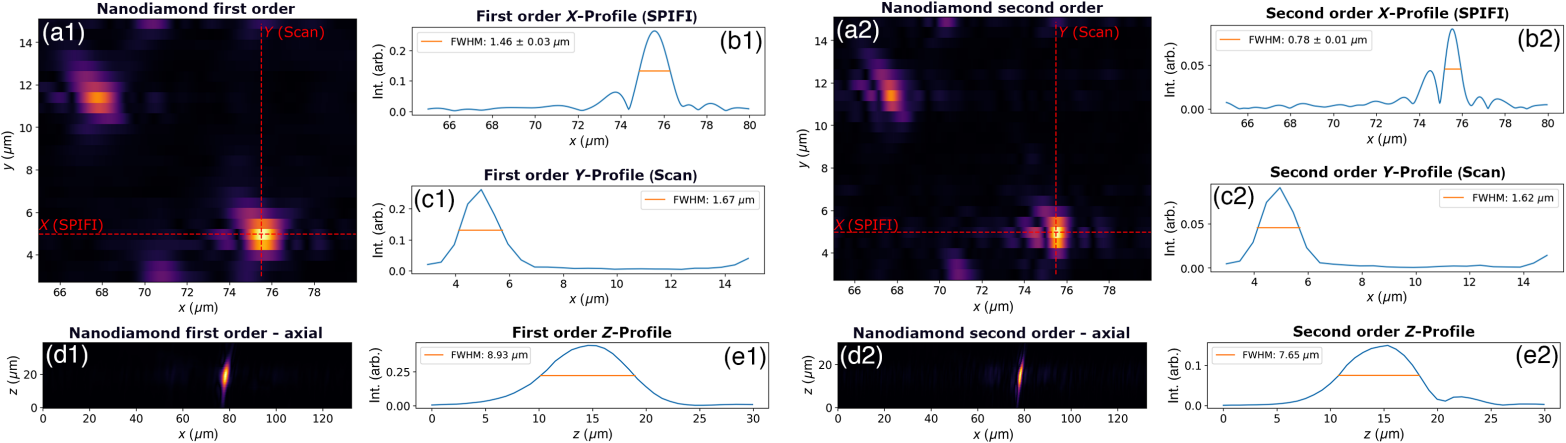
SPIFI images of the nanodiamond sample in the first order (a1) and the second order (a2). Line profiles across a nanodiamond are shown in X along the SPIFI axis for both orders (b1) and (b2), as well as in the scan direction Y (c1) and (c2). Axial images were taken in the X-Z plane (d1) and (d2) with profiles included in the Z direction (e1) and (e2).

Measuring the PSF is a common metric for system performance. The PSF was measured by imaging a sample with sub-diffractive fluorescent nanodiamonds spread across a microscope coverslip. The sample was produced by soaking the coverslip in a poly-l-lysine (0.1%) solution for 60 min, rinsing in de-ionized water, allowing it to dry, and then spreading 3  μL of the 1.5 ppm nanodiamond solution across the coverslip. The 140 nm nanodiamond (Adamas Nano NDNV140nmMd10ml) exhibits 2PEF under the 1035 nm pulsed laser, using the 209 fs duration pulse input into the microscope. Because SPIFI images are generated in the temporal frequency domain, a calibration is required to determine the relationship between a pixel’s blinking frequency on the mask and its position at focus. For this calibration, a USAF target was placed at focus, and light was collected onto a photodiode. The center-to-center distances of each bar of each element in group 6 were measured in frequency space and compared with their separation in space. This yielded a calibration of $1.32\times 10^{-8}\;\mu m/\mathrm{Hz}$ with an uncertainty of $\pm 2.54\times 10^{-10}\;\mu m/\mathrm{Hz}$. With this calibration, SPIFI images of the nanodiamond sample demonstrate a PSF FWHM of 1.46±0.03  μm in the first order and 0.78±0.01  μm in the second order. Along the scan axis where the resolution is dependent on the transverse focusing of the line cursor and the movement resolution of the sample stage, the FWHM values for the first and second orders were 1.67 and 1.62  μm, respectively. In the axial (Z) direction, the nanodiamond FWHM values were 8.93  μm for the first order and 7.65  μm for the second order. The resolution measurement images and profiles are shown in Fig. 8. The average power following the objective onto the sample for these images was ∼200  mW (20 nJ pulse energy). Images were taken through the coverslip on the back surface with the nanodiamonds for aberration correction in the objective.

For the scan direction (y-axis), it is important to consider the limitations of the sample motion stage in this analysis. The Newport LTA-HS specifications indicate the following parameters for typical performance: minimum incremental motion 0.10  μm, accuracy (typical) ±2.2  μm, bi-directional repeatability (typical) ±0.30  μm, and uni-directional repeatability (typical) ±0.10  μm. Guaranteed parameters are higher than these typical parameters, and additional errors may be present due to the stage to which the motor is mounted. To find nanodiamonds in an initial scan and return to them for longer exposures, we found that 0.50  μm steps were the minimum step size that we could use to reliably return to nanodiamonds for scanning and smaller step sizes often did not yield any apparent movement in the image through multiple steps. For these reasons, the y-axis profile PSF measurements of the nanodiamond should be considered an estimate. With regards to the performance on the SPIFI axis, those PSF measurements are not dependent on the motor stage motion; they are only dependent on the SPIFI modulation mask and the optics that generate the line cursor and image it to the sample. The profiles in the axial (Z) direction were corrected for the index of refraction change through the coverslip as in the z-sectioning analysis in Fig. 10.

The MTF measurement was done by placing a mirror at the sample plane and a dichroic (Semrock LF635) between the tube lens and objective lens. The ∼2% reflection of the 1035 nm light enables epi-direction imaging with a focusing lens onto a camera (AmScope MU1000). First, a calibration must be taken by either measuring the pixel width of a sample with known width or moving a sample a known distance and measuring the pixel shift. With a camera pixel calibration to the distances at the sample plane, the SPIFI mask was then re-mounted from the DC motor for imaging to a stepper motor (Trinamic QSH2818). Using a Python script to control the stepper motor and to retrieve image data from the camera, a line profile from the average intensities across the short-axis of the modulated cursor was recorded over 400 motor steps through the SPIFI modulation pattern. Each line profile was normalized against the unmodulated line beam intensity, and the FFT for each profile was taken.

**Fig. 9:**
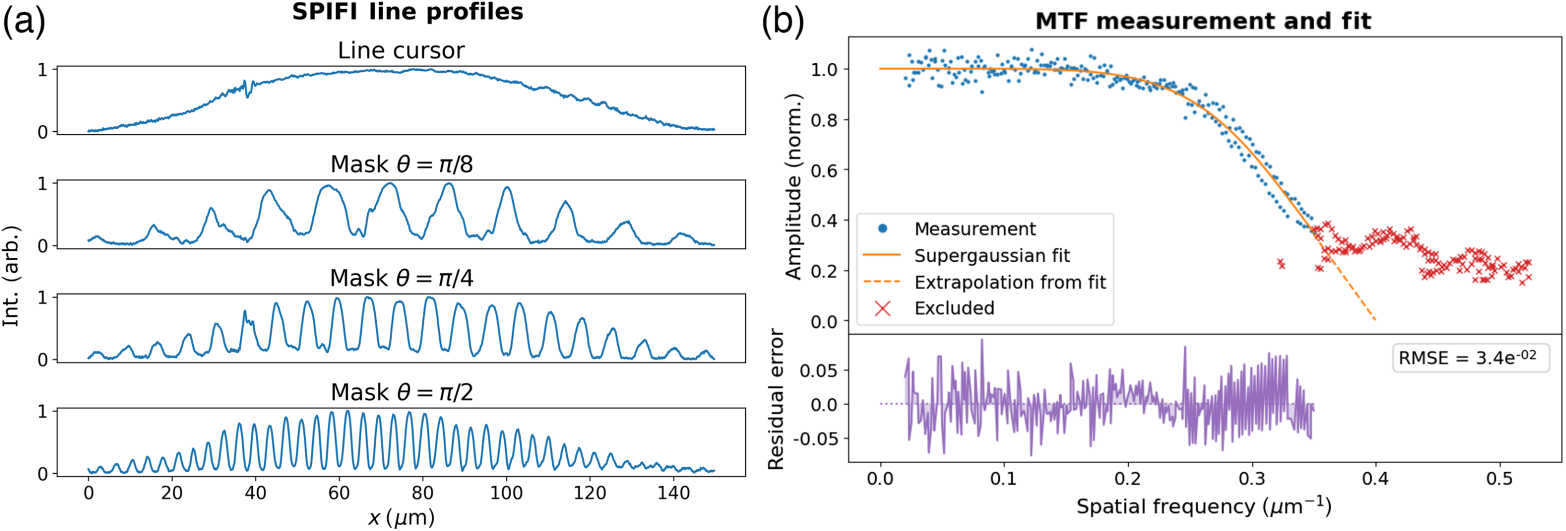
(a) Profiles showing the unmodulated line cursor and the line cursor with SPIFI modulation at a few select rotations. (b) Top: measured MTF data, excluded points, and the super-Gaussian curve of best fit with extrapolation to zero. Bottom: residuals between the curve of best fit and the measured data. Residuals appear biased positive near the inflection point, indicating a slight mismatch for the fit function. Residuals in other areas appear to be mostly random noise about zero.

Using the SciPy peak finding algorithm,^46^ the peak intensities and spatial frequencies for each line profiles were measured. The results are shown in Fig. 9. A subset of the resulting data points follows a profile as seen in analytic MTFs for SPIFI.^26^ The FFT peaks for this subset of data points are clear, single frequencies. At higher spatial frequencies, the FFTs do not show a single peak frequency, rather broad regions of multiple peak frequencies. The line profiles for these also show periods of no resolvable modulation and periodic modulations similar to beat frequencies. To fit a function to the data, these points were excluded from the fit. The cause of this is imperfect focusing of the line cursor onto the SPIFI mask, which yields aliasing and beating of frequencies, as seen in Ref. 42.

**Fig. 10:**
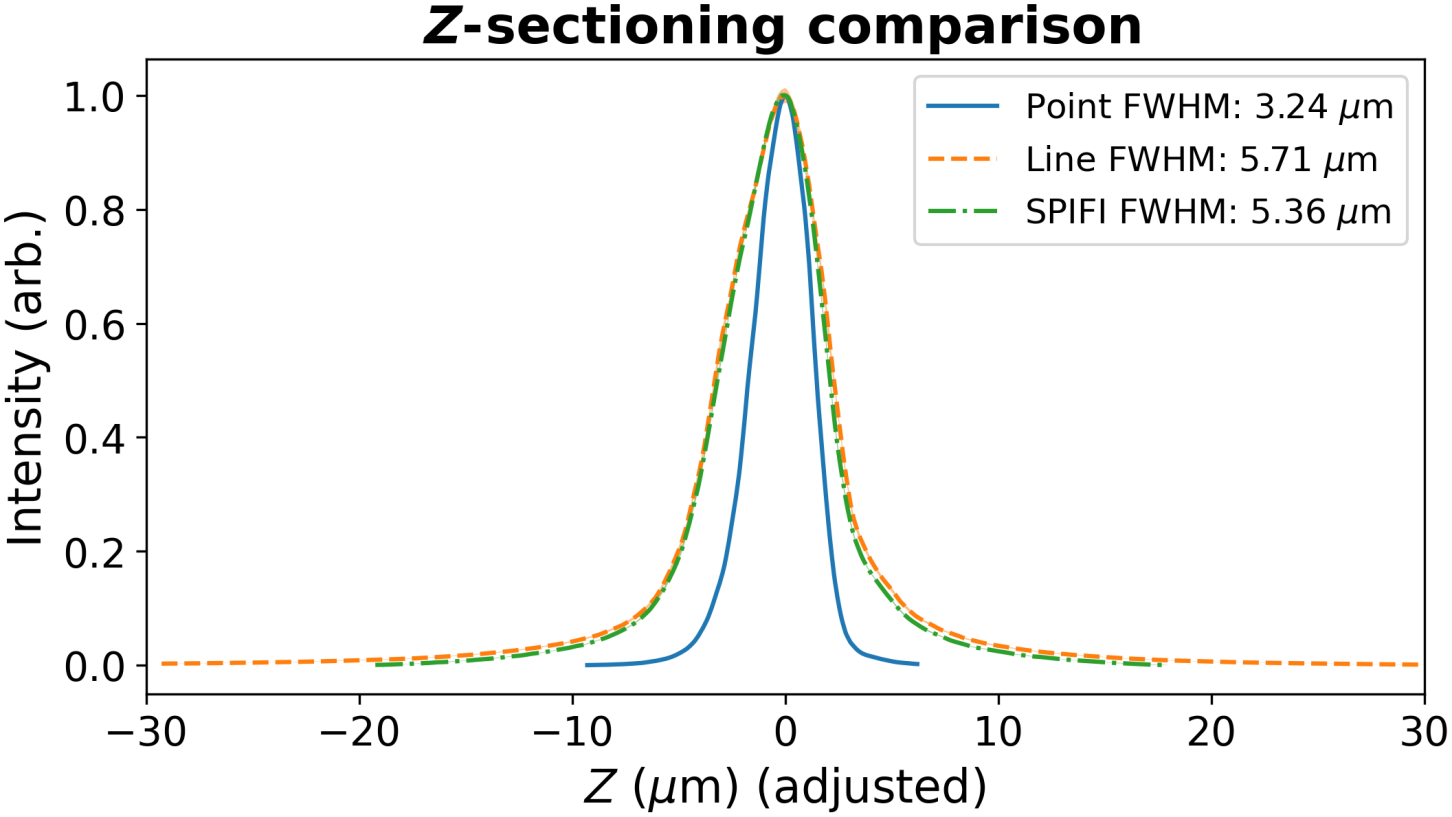
A comparison of z-sectioning with a point focus, line focus, and modulated SPIFI line focus. Each dataset was aligned using a cross-correlation measurement for comparison. As expected, a point focus yields the finest sectioning, while SPIFI sectioning is marginally better than that of a line focus.

The data were fit to a super-Gaussian of the form $f(x)=a\;\exp[-2(x/b{)}^{c}]$ using SciPy. The parameters of best fit and standard deviations for a, b, and c were $1.00\pm 3.15e^{-3}$, $3.86e^{-1}\pm 2.29e^{-3}$, and $6.24\pm 1.71e^{-1}$, respectively. The residuals between the fit line and the data points are also shown in Fig. 9 and show a root-mean-square error of $3.4e^{-2}$. The NA can be evaluated with the MTF measurement by taking the cutoff frequency $f_{c}$ as the highest spatial frequency (not including the excluded data points) via the equation $\mathrm{NA}=\lambda_{0}f_{c}$. The highest non-excluded spatial frequency in this data set is $0.35\;\mu m^{-1}$, yielding an NA of 0.36. At this NA, the FWHM of the PSF for a point-focus in linear imaging such as single photon excitation is calculated by $0.51\lambda_{0}/\mathrm{NA}$, yielding 1.46  μm. For a second order process, such as the 2PEF exhibited in the nanodiamond images in Fig. 8, the FWHM for a point-focus is calculated using $0.64\lambda \sqrt{\ln\;2}/\sqrt{2}\mathrm{NA}$,^47^ yielding an FWHM of 1.08  μm. In SPIFI, the spatial frequency cutoff support for each order is independent of modality (linear and non-linear).^26^ The first order SPIFI result in Fig. 8 at this NA yields an FWHM of 1.46  μm, whereas the second order image in Fig. 8 produces a PSF FWHM of 0.78  μm.

Extrapolating the near-linear decline of the MTF fit line enables an estimate of the cutoff frequency with better cursor modulation. This extrapolated line intersects with the x-axis at a spatial frequency of $∼4\;\mu m^{-1}$, indicating a possible NA of 0.41 and a PSF FWHM of 1.28  μm. Further improvement would be possible using a SPIFI mask with finer feature sizes, provided the cursor was focused sufficiently tightly, such that the diffracted orders fill the entrance pupil of the objective. With this mask, the spacing between the ±1 orders was at most 4.5 mm, and the objective pupil diameter was 6.75 mm.

The z-sectioning (axial-sectioning) of the SD-SPIFI microscope was examined and compared with that of a point focus and that of an unmodulated line cursor. This was done by scanning the focus through a coverslip that generates third harmonic light at the interfaces.^48^^,^^49^ First scanning through the front and back surface yields a scanned distance between third harmonic generation (THG) at each interface. This can be compared with the actual coverslip thickness measured by calipers to find a factor to adjust the z-axis due to the focusing angle changing when travelling through the glass. The measured coverslip thickness was 170  μm, and the surface-to-surface focusing distance was measured as 220  μm, yielding an adjustment factor of 0.77. Following this, scans were taken at a fine step resolution through the back surface of the glass to measure a profile of THG generation. The results of this for the point focus, line focus, and SPIFI modulated line are shown in Fig. 10. SPIFI yields a z-sectioning result broader than for a point focus but marginally better than that of a regular line cursor.

**Fig. 11:**
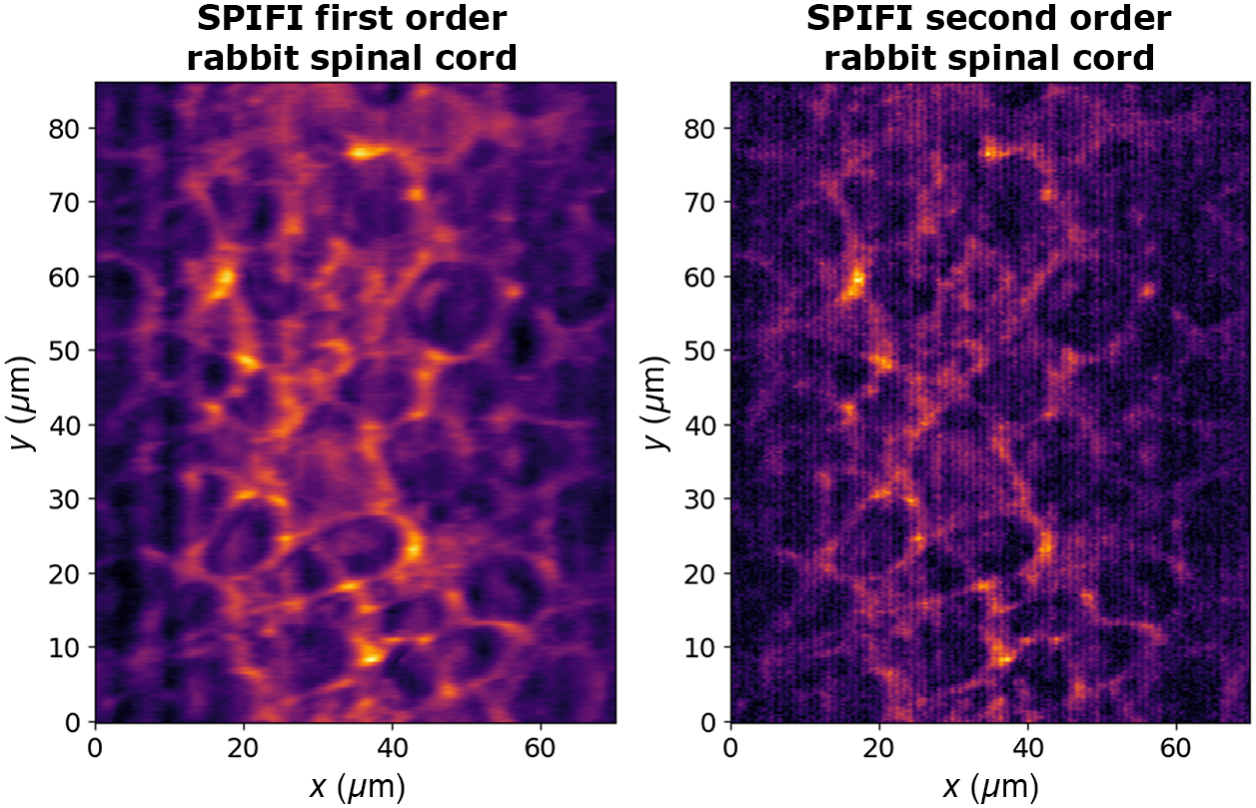
First and second order 2PEF SPIFI images of a dyed rabbit spinal tissue sample. Incident average power was ∼60  mW. The lower SNR of the second order image makes ringing artifacts apparent along the frequency (x-axis).

Images of a dyed rabbit spinal cord tissue sample were taken with SPIFI using 2PEF with the 209 fs pulse input into the SPIFI microscope. First and second order images are shown in Fig. 11. Images were taken with 60 mW of average power (6 nJ pulse energy) incident on the sample and 30 averages of the FFTs calculated from a 15.5 ms exposure signal. No photon counting was done for these images, and each FFT was computed from the analog PMT signal. The second order SPIFI image has the benefit of enhanced resolution with the smaller PSF from Fig. 8 but lower SNR. This lower SNR makes ringing artifacts more apparent in the image. These ringing artifacts (vertical lines) can be related back to the PSF measurements in which larger side lobes are visible, especially in the second order. These lobes in the PSF manifest as these line artifacts in images as the PSF is convolved with the object. SNR improvements can be made by implementing photon counting for future work, enabling longer exposure times and greater sample averaging. The spinal cord sample was purchased from Happy Science Co.; it has a sample thickness of 7  μm and is stained using hematoxylin & eosin (H&E). At this wavelength ($\lambda_{0}=1035\;\mathrm{nm}$), the Eosin in the stain can absorb two photons for excitation and subsequent fluorescence. The samples were prepared by the manufacturer by collecting the sample material, dehydration, staining with H&E, embedding in parrafin wax, slicing to 7  μm, mounting to the slide, sealing, and affixing the cover slip and label. The SPIFI axial sectioning demonstrated in Fig. 10 is near the total thickness of this sample, indicating that the image contains fluorescence from nearly the full 7  μm thickness and minimal or no bulk was passed through prior to fluorescence.

**Fig. 12:**
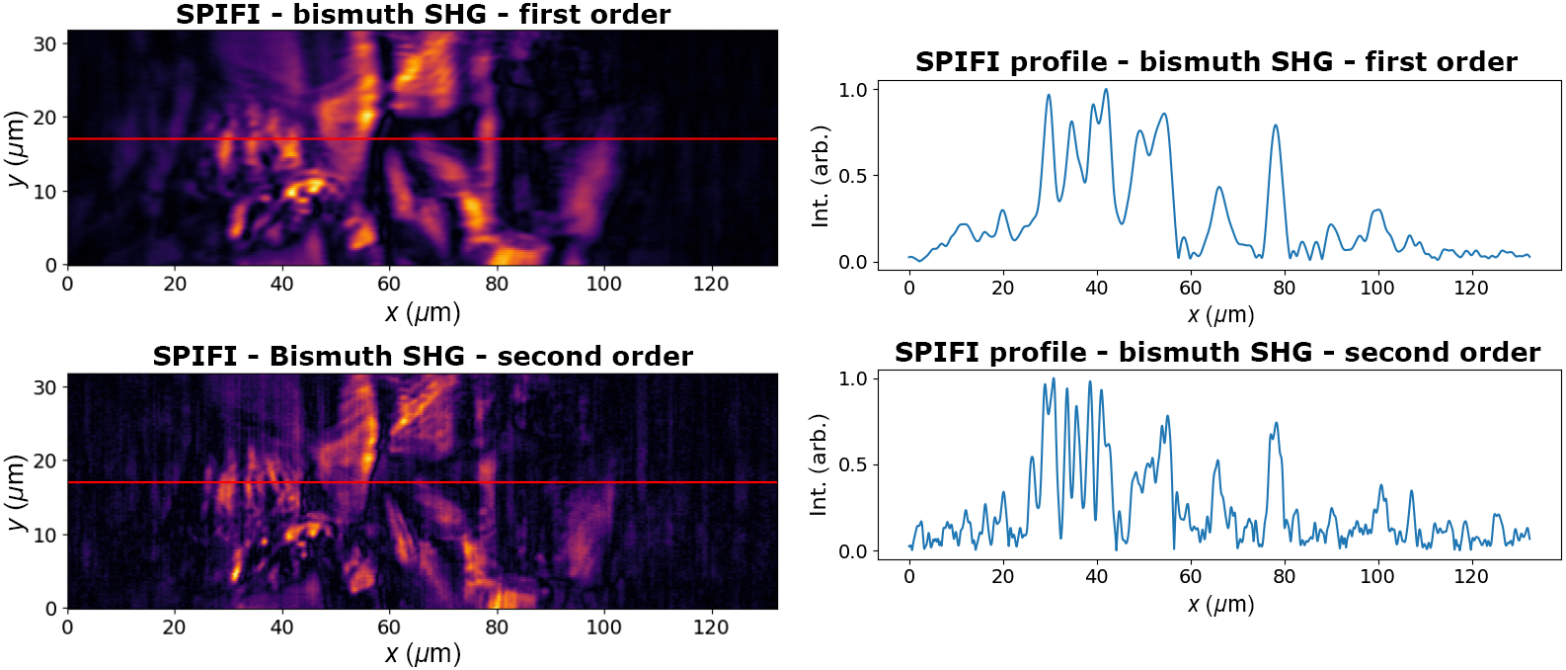
SHG signal of a bismuth halide sample in the first and second SPIFI orders. Profiles from the red lines are shown to emphasize the resolution enhancement in the second order.

**Fig. 13:**
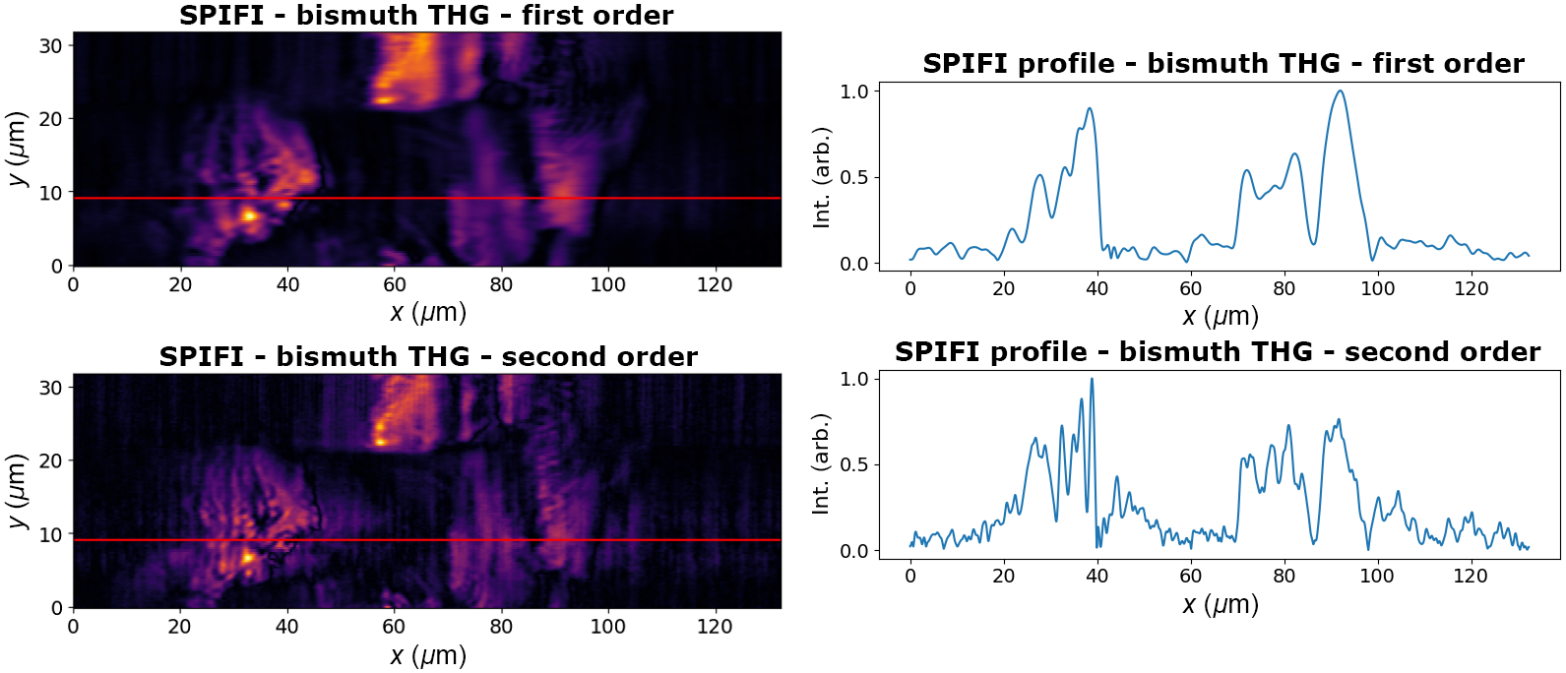
THG signal of a bismuth halide sample in first and second SPIFI orders. Profiles from the red lines are shown to emphasize the resolution enhancement in the second order.

SPIFI is also compatible with harmonic generation. The surface of a Bismuth Halide thin film sample, <1  μm thick, was imaged with both SHG and THG. In both of these modalities, the improvement in resolution in the second order is apparent. Along one line capture on the SPIFI axis, marked in red in Figs. 12 and 13, the comparison between first and second orders shows significantly more detail and discernment in features. There is, however, lower SNR due to the lower amplitude of the higher order SPIFI signals. The SHG image was taken with 15 mW of average power (1.5 nJ pulse energy) incident on the sample, and the THG image was taken with 24 mW of average power (2.4 nJ pulse energy) on the sample. The same 209 fs duration pulse was the input into the SPIFI microscope. This hybrid organic-inorganic semiconductor sample was prepared by dissolving the precursors, spin-coating onto a substrate, and annealing on a hot plate. This yields a polycrystalline film that can show the image differences between the SHG and THG modalities.

## 4 Next Steps

All image results presented here are from raw PMT signals acquired on an oscilloscope with the only post processing being wobble correction, timing alignment, and FFT averaging. FFT averaging is used because the shot-to-shot variation in raw PMT signals is Poissonian in nature and simple averaging does not improve signal results—one SPIFI trace may have a PMT detection signal at one time step, and the next SPIFI trace may not. Averaging signals that sometimes contain signal photons at certain time steps and sometimes do not yields an overall reduction in total signal and worsens SNR. Signal improvements can instead be realized using a longer exposure additive signal approach, such as with a digitized photon counting approach. Using a threshold to increment PMT signal counts in discrete time bins that correspond with incident excitation pulses reduces extraneous noise and builds up a signal over time, which reduces SNR and enables higher exposure times. This improvement in SNR will make third and fourth order imaging more reliable to potentially get a nearly 4× resolution enhancement. Currently, third and fourth order images are obtainable from the data presented here, but due to noise, the resolution results are not repeatable enough to report.

## 5 Conclusion

We have presented a cascaded SPIFI system that modulates in both the WD and the SD. By modulating only the WD, second order dispersion compensation was optimized through the full imaging system by moving a translation stage and viewing a real-time signal. This result was confirmed with a full pulse characterization technique, dispersion scan, and the WD-SPIFI optimization was readily and quickly repeated following any system changes.

With the SD SPIFI system, we demonstrated enhanced resolution in multiple multiphoton modalities (2PEF, SHG, and THG) with a PMT and no use of photon counting or post-processing beyond averaging and standard SPIFI image generation with FFTs. The PSF of the system was measured using a sub-diffractive nano-diamond, which also directly measured the PSF enhancement for second order SPIFI. The MTF was also measured using the spatial frequency modulation of the SPIFI mask, and the effective cutoff frequency matched the result from the PSF measurement.

## Code, Data, and Materials Availability

Data, code, and materials information relevant for this article are not publicly available but can be provided upon reasonable request.
